# Prevalence of Internet Addiction and Its Association With Lifestyle Factors Among University Students: A Pilot Study in Eastern India

**DOI:** 10.7759/cureus.85061

**Published:** 2025-05-29

**Authors:** Jayanti Mishra, Manas Ranjan Behera, Priyadarsini Samanta, Sonali Kar, Pranab Mahapatra, Jigyansa Ipsita Pattnaik, Rituparna Mitra

**Affiliations:** 1 Physiology, All India Institute of Medical Sciences, Bhubaneswar, Bhubaneswar, IND; 2 Public Health, Kalinga Institute of Industrial Technology (Deemed to be University), Bhubaneswar, IND; 3 Physiology, Kalinga Institute of Medical Sciences, Bhubaneswar, IND; 4 Community Medicine, Kalinga Institute of Medical Sciences, Bhubaneswar, IND; 5 Psychiatry, Kalinga Institute of Medical Sciences, Bhubaneswar, IND; 6 Research and Development, Kalinga Institute of Medical Sciences, Bhubaneswar, IND

**Keywords:** diet, internet addiction, lifestyle, non-vegetarian, physical activity, prevalence

## Abstract

Background: Internet addiction (IA), identified as compulsive use of the internet, is characterized by an individual's unwillingness to cease internet usage despite adverse impacts on their mental, physical, and psychological well-being. Recent studies reporting a high prevalence of IA among university students have also found an association of IA with factors such as academic performance, depression, obesity, substance abuse, unhealthy diet, and more. Among all the factors, lifestyle factors were the least explored. Hence, this study was carried out to find the prevalence of IA and its association with diet preferences and physical activity.

Methods: This was a cross-sectional pilot study conducted among university students in eastern India. We included 100 enrolled students from different courses at the university. A standardized tool was prepared, including socio-demographic and lifestyle factors, especially diet and physical activity. Young’s Internet Addiction Test (IAT) scale was used to estimate the IA among the students.

Results: The mean age of the students was 19.1 ± 1.40 years, with a male majority of 55 (55%). Almost half of the students were studying engineering (48, 48%). Most students were non-vegetarian (58, 58%) and exercised less than three days per week (54, 54%). IA was found in 71 (71%) of students. Out of all students, 59 (59%) had mild addiction, 11 (11%) had moderate addiction, and one (1%) had severe addiction to the internet. Non-vegetarian food preference was significantly associated with the severity of IA. We found no statistically significant association between age, gender, obesity, physical activity, or courses enrolled with IA.

Conclusions: Our study revealed a high prevalence of IA among university students, which was significantly associated with eating preferences. Although students with IA were less involved in physical activity, it was not statistically significant. Further studies can generate more evidence on IA in the eastern region of India.

## Introduction

The internet is a primary medium reshaping young education and social interaction in this century. This form of communication has developed into perhaps the greatest beneficial and sophisticated technological innovation. The internet provides access to several services, including emails, the World Wide Web, and platforms such as Facebook, Instagram, and Telegram [1,2].

Internet addiction (IA), identified as compulsive use of the internet, is characterized by an individual's unwillingness to cease internet usage despite adverse impacts on their mental, physical, and psychological well-being [3]. IA, characterized by an individual's inability to regulate their internet usage, is considered a significant mental health issue due to its potential to induce stress and impede functionality in daily activities [4]. The worldwide prevalence of IA was reported at 7%, with variations observed across different regions [5].

In the past few decades, developing nations such as India have experienced an exponential rise in internet usage. The internet's easy accessibility and reasonable price have broadened its user base from professionals to the general population. The internet is a worldwide platform for disseminating and exchanging information at minimal expense. Using internet resources and their consequences has emerged as a contentious topic. On the one hand, it is the most significant and essential requirement of contemporary individuals; on the flip side, individuals exhibit excessive dependence on it. Studies indicate that regular internet usage contributes to several psychological and mental diseases [6,7].

A study involving medical students revealed that 65% of participants were average users, 12% were potential IA, and 0.4% had IA, with a notably high average duration of internet use [8]. Multiple studies conducted by Anand et al. at multiple time points revealed that within the cohort of medical students, the occurrence rates of mild IA ranged from 27 to 37%, moderate IA from 9.7 to 33.1%, and severe IA from 0.4% to 3.1%. The studies showed that the prevalence of IA increased significantly in the past decade [9-11]. In a study of university students, the severity of IA was reported and revealed that the mild, moderate, and severe IA rates were 30%, 16%, and 0.5%, respectively. Among engineering students, these rates were 27.1%, 9.7%, and 0.4%, indicating a strong correlation between problematic internet use, disrupted sleep patterns, and depressive symptoms [12,13]. This aligns with recent data demonstrating that the prevalence of IA has risen in recent years [14].

Lifestyle is an important factor among adolescents and youth that shapes their future lives. Although economic conditions and the availability of choices determine the lifestyle of youth, research findings suggest an association with IA. A study on adolescents found that IA was significantly associated with six important lifestyle domains: spirituality, health responsibility, nutrition, physical activity, interpersonal relations, and stress management [15]. Similar findings were observed in other studies on university students, adolescents, and youths of developed nations [16]. Studies ascertaining association in developing nations, and more so in the resource-poor states, are limited. Therefore, this study was carried out with the specific objectives of finding the prevalence of IA and its association with diet and physical activity.

## Materials and methods

This was a cross-sectional pilot study conducted among university students in eastern India. We included 100 enrolled students from different courses at the university. A semi-structured questionnaire (including both closed and open-ended questions) was used to collect responses from the students. The questionnaire included socio-demographic factors such as age in completed years, gender, and monthly family incomes in rupees, along with educational details. Weight in kilograms and height in meters were measured using a standard and calibrated weighing scale and stadiometer, respectively. Lifestyle factors, especially preference for vegetarian or non-vegetarian food and frequency of physical activity per week, were collected.

Young’s Internet Addiction Test (IAT) scale [17] was used to estimate the IA among the students. The IAT scale has 20 items based on the Diagnostic and Statistical Manual of Mental Disorders-IV, and each item was rated on a Likert scale from 1 (not at all) to 5 (always). The 20 items include the personal aspect of the individual with respect to his/her social life, routine activity, sleep pattern, productivity, and feelings. Minimum and maximum scores ranged from 20 to 100. The total score was calculated by adding each score of 20 items and further classified into no addiction (20-30), mild addiction (31-49), moderate addiction (50-79), and severe addiction (80-100). The IAT scale was a valid and reliable scale, with internal consistency (Cronbach’s alpha around 0.9) and test-retest reliability (r = 0.8) [18,19].

Students were approached at the end of their scheduled class and requested to participate in the study. After describing the study in detail and obtaining written informed consent, students filled out the self-administered questionnaire. The questionnaire took an average of eight minutes to complete by the students. A convenience sampling (contacted during scheduled class in person) method was used to select the students.

Data collected were entered in Microsoft Excel software (Microsoft® Corp., Redmond, WA) and imported to Statistical Product and Service Solutions (SPSS, version 27; IBM SPSS Statistics for Windows, Armonk, NY) for further analysis. Categorical variables were expressed in frequency and percentages, while the continuous variables were expressed in mean and standard deviation. The association between two categorical variables was calculated using the chi-square test, and the comparison of means was performed using the ANOVA/Kruskal-Wallis test. P value <0.05 was considered statistically significant. The institutional ethics committee approved the study.

## Results

The mean age of the students was 19.1 ± 1.40 years, with a male majority of 55 (55%). Almost half of the students were studying engineering (48, 48%), followed by pharmacy (33, 33%), and dental science (14, 14%). Most students were non-vegetarian (58, 58%) and exercised less than three days per week (54, 54%) (Table 1).

**Table 1:** Baseline characteristics of the study participants (N = 100)

Characteristics	Frequency (%) or Mean (SD)
Gender	
Male	55 (55.0%)
Female	45 (45.0%)
Age in completed years	19.08 (1.40)
Height in centimeters	158.09 (12.05)
Weight in kilograms	56.98 (16.65)
Body mass index in kg/m^2^	22.71 (5.68)
Name of the Course	
Bachelor’s in technology	48 (48.0%)
Pharmacy	34 (33.0%)
Bachelor’s in dental science	13 (14.0%)
Other Allied	5 (5.0%)
Monthly family income	198,325.58 (300,639.96)
Food Habit	
Vegetarian	42 (42.0%)
Non-vegetarian	58 (58.0%)
Frequency of Exercise per Week	
< 3 days per week	54 (54.0%)
>= 3 days per week	46 (46.0%)

**Figure 1:**
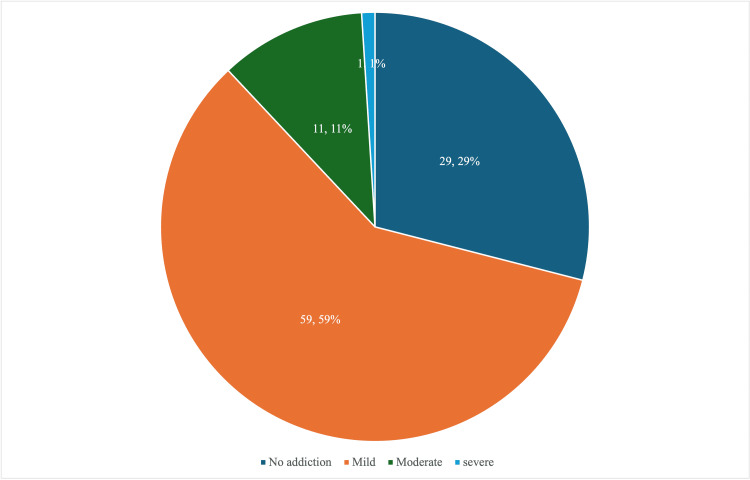
Internet addiction among the study participants (N = 100)

IA was found in 71 (71%) of students. Out of all students, 59 (59%) had mild addiction, 11 (11%) had moderate addiction, and one (1%) had severe addiction to the Internet (Figure 1). 

Since only one student belonged to the severe category, we considered the moderate and severe addiction groups combined for performing statistical associations. Although it was observed that the body mass index (BMI) of the students increased as the severity of addiction increased, the change was not statistically significant (p-value = 0.095). Although males and engineering students were more addicted than females and medical/allied sciences students, the association was not statistically significant (p-value > 0.05). Family income, age, and physical activity per week were not associated with the severity of IA. A non-vegetarian diet was more prevalent among students who were addicted to the Internet (p-value < 0.001) (Table 2).

**Table 2:** Association between lifestyle factors with internet addiction (N = 100)

Variables	Internet addiction			
	No addiction	Mild	Moderate/severe	P value
Gender				
Male	15 (51.7%)	32 (54.2%)	8 (66.7%)	0.670
Female	14 (48.3%)	27 (45.8%)	4 (33.3%)	
Age in years	19.24 (1.72)	18.85 (1.05)	19.83 (1.80)	0.062
BMI in kg/m^2^	20.79 (3.67)	23.45 (6.01)	23.70 (7.20)	0.095
Course Enrolled				
Engineering	9 (31.0%)	33 (55.9%)	6 (50.0%)	0.088
Medical and allied	20 (69.0%)	26 (44.1%)	6 (50.0%)	
Family income in thousands per month	118.33 (122.59)	233.17 (354.52)	209.10 (280.74)	0.303
Food Habit				
Vegetarian	21 (72.4%)	16 (27.1%)	5 (41.7%)	<0.001
Non-vegetarian	8 (27.6%)	43 (72.9%)	7 (58.3%)	
Exercise				
< 3 days per week	16 (55.2%)	32 (54.2%)	6 (50.0%)	0.954
>= 3 days per week	13 (44.8%)	27 (45.8%)	6 (50.0%)	

Table 3 compares the raw IA scores between different at-risk categories. The IA scores were comparable among the age, gender, BMI, course enrolled, and exercise frequency categories (P value > 0.05). The mean score among students consuming non-vegetarian food was significantly higher compared to vegetarian students (P value = 0.007).

**Table 3:** Comparison between internet addiction (IA) scores with respect to different risk factors

Variables	Mean	SD	P value
Age			
≤ 20 years	35.58	14.21	0.685
> 20 years	33.75	17.61	
Gender			
Male	35.45	16.46	0.943
Female	35.24	12.04	
Course Enrolled			
Engineering	36.68	13.04	0.384
Medical and allied	34.13	15.88	
BMI			
Underweight	32.76	15.86	0.054
Normal	34.00	12.15	
Overweight	36.71	12.59	
Obese	48.25	21.18	
Exercise			
< 3 days per week	36.20	15.49	0.533
≥ 3 days per week	34.37	13.51	
Food Habit			
Vegetarian	30.76	17.38	0.007
Non-vegetarians	38.69	11.17	

## Discussion

The enhanced accessibility, broader acceptability, and rapid expansion of the World Wide Web have resulted in multiple impacts on users' social lives and psychological states, particularly young adults. This pilot study aimed to assess the prevalence of ID among university students and the association of its severity with a few selected parameters in the eastern region of India.

The current study revealed the rate of IA at 71%. The severity of IA was classified as mild, moderate, and severe among 59%, 11%, and 1% of participants, respectively. Conversely, a survey done in Nepal indicated that 51% of the participants had a moderate-to-severe level of IA. The difference can be attributed to the participation of younger people (less than 20 years) in the study [20]. A study in Kashmir by Bhat et al. indicated that the percentages of respondents experiencing mild, moderate, and severe IA were 42%, 29%, and 30%, respectively [21]. Goel et al. reported that 75% of the students were moderate users; among them, 25% were potential addicts, and 1% were classified as addicts [22]. A study conducted among medical and allied students at a prominent university in Saudi Arabia indicated a proportion of 65.21% [23]. Conversely, Gedam et al. reported a notably low frequency of IA at 20%. Nonetheless, the moderate and severe levels of IA, at 20% and 0.4% respectively, corroborated the current study findings [24]. The current study revealed that the proportion of IA among engineering students was higher compared to medical and allied sciences students, which was similar to the study conducted by Murarkar et al., which indicated a higher prevalence among engineering students [25]. 

Few studies found a significant negative association between physical activity and IA [15,16]. A study by Shakori et al. [15] included 407 students in their research and reported significantly less physical activity among students exposed to IA. They concluded that IA facilitates sedentary behaviours and students show little inclination towards physical activity. Another study by Ying et al. [16] reported a similar finding that students with IA were more sedentary, defined as more than three hours of sitting activity per day. Our study did not find a statistical association, which may be attributed to the geographical variation and current trends among university students.

Our study observed a significant association between a non-vegetarian diet and IA. To the best of our knowledge, only a few studies have compared non-vegetarian food preference with IA, while many studies have compared eating disorders/unhealthy food preference with IA. A study by Waheed et al. found a strong positive correlation between meat and fried food with IA among students of medicine and dentistry, which was similar to our study finding [26]. A study by Mohammad Johari et al. found that the severity of IA also affects the food choices that affect their sensory appeal and mood [27]. This supports our study finding, as in our study sample, that non-vegetarian food may be the food preferred by the students with problematic internet use. Few studies reported an association of eating disorders with IA [15,16], and a study by Knight et al. reported a preference for non-vegetarian food in students with eating disorders, which also supports our study findings [28].

The strength of the study was the use of a well-validated and reliable scale to measure IA. Our study has a few limitations, as the sample size was smaller, as it was a pilot study, and based on this study’s results, a larger study will be carried out. The generalizability of our study was also limited as it was carried out in a single university, which can affect the choices and preferences of the students.

## Conclusions

Our study revealed a high prevalence of IA among university students, which was significantly associated with eating preferences. Although a higher proportion of the students with IA were involved in less physical activity, we did not find any significant association. Future studies with larger sample sizes and proper methodology will provide insight into IA trends and explore the associated factors among students in the eastern region of India.
